# Developmental Patterns of Cognitive Function and Associated Factors among the Elderly in Taiwan

**DOI:** 10.1038/srep33486

**Published:** 2016-09-16

**Authors:** Ting-Yu Chen, Hsing-Yi Chang

**Affiliations:** 1 Graduate Institute of Life Sciences, National Defense Medical Center, Taipei, Taiwan; 2 Institute of Population Health Sciences, National Health Research Institutes, Miaoli, Taiwan; 3 Institute of Public Heath, National Yang-Ming University, Taipei, Taiwan

## Abstract

Previous research has discussed the factors associated with cognitive impairment, but the patterns of its development have been little described. Our aim was to examine long-term development of cognitive function and the related factors using longitudinal follow-up data. A group-based trajectory model and multinomial logistic regression were applied to identify trajectories and the associated baseline factors, and a mixed model was used to identify the time-varying factors associated with the trajectories. Three trajectories were identified: starting low and declining (30.8%), starting high and declining (51.8%), and high-stable (17.4%). These three trajectories were apparent at the beginning of the study and did not crossover during the study period. Smoking, diabetes, depression, and instrumental activities of daily living were significant variables for differentiating the starting high and declining group from the high-stable group. Similar patterns and emotional support as a contributing variable were observed in the starting low and declining group. Physical activity, self-rated health, cardiovascular diseases, depression score, physical function, and social support were related to the trajectories over time. Impaired physical function, cardiovascular diseases, depression symptom, and poor social support in late life may be potential risk factors affecting the decline of cognitive function. Preventive strategies should be designed accordingly.

According to the World Health Organization, the world population aged 60 years and older is expected to reach more than 2 billion in 2050^1^, double the current number. With the aging population growing rapidly, the number of people with dementia is increasing. As of 2015, 46.8 million elderly people were living with dementia, with 9.9 million new cases every year worldwide, and the prevalence of dementia is expected to increase to about 74.7 million cases in 2030 and 131.5 million in 2050^2^. The high prevalence and incidence modifies survival and increases the risk of death^3^. In the United States, one of three seniors dies with Alzheimer’s or another form of dementia^4^. The estimated median survival times for Alzheimer’s disease and vascular dementia are 7.1 and 3.9 years, respectively^5^.

The growing number of people with dementia will increase the disability burden and will bring enormous costs from long-term care. In 2015, the total global societal costs of dementia were estimated to be US$818 billion, and more than 80% costs were attributed to informal care and residential care^2^. Caregivers of people with dementia may experience higher levels of stress^6^, psychological morbidity^7^, and impaired physical health^8^; they even have increased risk of mortality^9^. In addition, no treatments have been developed to cure dementia or alter its progressive course, and research identifying modifiable risk factors for dementia is limited. Dementia is an important health and care issue encountered throughout the world.

Taiwan is one of the most rapidly aging societies, and the prevalence of dementia in people aged 65 years and over is expected to increase from 1.02% of the total population in 2013 to 5.08% in 2060^10^. Given this steep increase, a model for determining the causes underlying cognitive dysfunction should be established to delay the development of dementia and reduce its social costs. Many studies have investigated possible factors affecting cognitive function^11^,^12^,^13^,^14^,^15^,^16^, such as cardiovascular disease^14^, obesity^17^, and physical activity^18^, among others. However, little research has examined the development of age-related cognitive impairment in the general population. Therefore, our aim was to explore the developmental trajectories of cognitive impairment in community-dwelling elderly adults and to identify the factors associated with it, using a nationally representative survey followed for 15 years.

## Methods

### Settings and participants

This study is a secondary data analysis utilizing data from the Taiwan Longitudinal Study on Aging (TLSA), a nationally representative investigation first conducted by the Health Promotion Administration, Ministry of Health and Welfare of Taiwan in 1989^19^. The prospective survey used a three-stage equal probability sampling design to understand the socioeconomic status and the physical and emotional health of the elderly in Taiwan. Fifty-six townships were first selected from 331 non-aboriginal areas. Next, blocks or lins were cluster sampled in each of the selected townships. Finally, individuals aged 60 or over were randomly sampled from each of the selected blocks. This process resulted in 4049 elderly subjects, with a survey response rate of 92% in the initial sample^20^. Follow-up surveys were conducted in 1993, 1996, 1999, 2003, and 2007^19^. Prior to 2007, people took part in the survey after giving verbal consent only, but in 2007, signed consent forms were collected by the Health Promotion Administration, Ministry of Health and Welfare of Taiwan.

**Figure 1:**
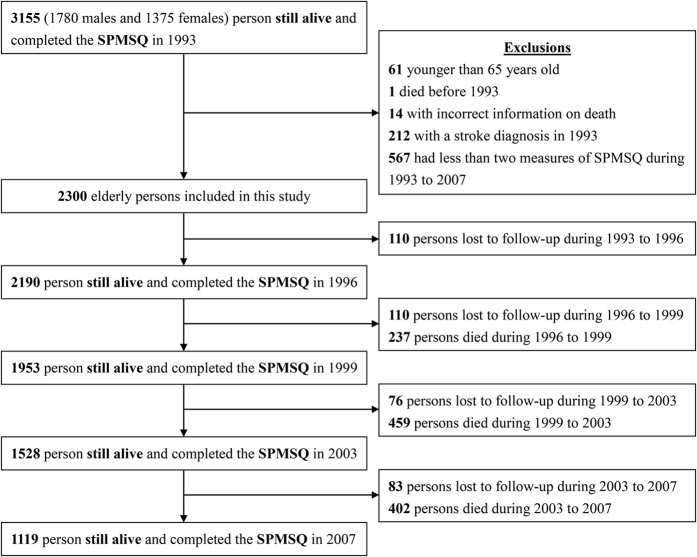
The flowchart of sample selection. Data source: Taiwan Longitudinal Study on Aging. SPMSQ = Short Portable Mental Status Questionnaires, with a score range of 0–5.

For the current study, we used data collected from 1993 to 2007, since the cognitive function measurement was implemented in 1993. All the selected participants or proxies speaking on their behalf were interviewed. A proxy could only be used if the participant (1) was severely ill or too frail to answer questions; (2) had cognitive problems; (3) had speech or hearing problems; or (4) had dementia and could not answer the factual questions in the TLSA study. The proxies did not undergo assessment of cognitive function and are therefore excluded from our analysis. We analyzed data for 3155 persons whose cognitive function was measured in 1993. We further excluded subjects under 65 years of age (n = 61); those who died before the 1993 survey began (n = 1), had incorrect information on death (n = 14), or had a stroke in 1993 (n = 212); and those with fewer than two assessments of the Short Portable Mental Status Questionnaires (SPMSQ) score (n = 567) during 1993 to 2007. A total of 2300 elderly subjects were included in the analysis (Fig. 1). This study was approved by the ethical committee of the National Health Research Institutions.

When we initiated the study, the TLSA was publicly available, and we could use the data directly. Since 2014, the use of secondary data has required ethics approval. We obtained the ethics approval from the Internal Review Board of Academic Sinica. All methods were carried out in accordance with relevant guidelines and regulations.

### Assessment of cognitive function

SPMSQ is a screening test for cognitive dysfunction^21^. We used a shorter form based on SPMSQ, which included only five questions. The total Cronbach’s alpha value of the five items was 0.63 in our population, indicating that the internal consistency was acceptable. These five items were validated by a Chinese version of the Mini-Mental State Examination^22^ and were used in the Asset and Health Dynamics among the Oldest-Old Study as an instrument of cognitive function^23^,^24^. A longitudinal study in Taiwan also used the five items as a tool to detect cognitive impairment^25^. The participants were asked the questions “What is today’s date (including month, day, and year)?”; “What is the day of the week?”; “What is your home address (or where are you)?” and “How old are you?”. They were also asked to subtract 3 from 20 for a total of four consecutive times. Each correct answer was given 1 point, and the range of the total score was 0 to 5, with 0 representing all five items being answered incorrectly. Individuals who completed SPMSQ at least twice were included.

### Covariates

We used the baseline age, sex, and education level for analysis. Other variables, including health status, health behaviors, physical function, and social supports, were obtained in 1993, 1996, 1999, 2003, and 2007, and were thus analyzed as time-varying variables.

Age was regarded as a continuous variable and was dichotomized into 65–74 and ≥75 years old, as shown in the descriptive analysis. Sex was a categorical variable coded as 1 (male) or 2 (female). Education level was defined as (1) illiterate, (2) uneducated but literate, (3) primary, and (4) secondary or above based on the number of years of formal education a person completed.

Health-related variables included body mass index (BMI), self-rated health status, chronic diseases, and depression symptom score. Self-reported height and weight data were used to calculate BMI (kg/m^2^), and we furthermore divided the participants into underweight (BMI < 18.5 kg/m^2^), normal weight (18.5 ≤ BMI < 24 kg/m^2^), overweight (24 ≤ BMI < 27 kg/m^2^), and obese (BMI ≥ 27 kg/m^2^) subgroups based on the criteria of the Ministry of Health and Welfare in Taiwan^26^. Health status was self-rated on a 5-point rating scale, with 5 = excellent, 4 = good, 3 = average, 2 = poor, and 1 = very poor. We regrouped the health status into three subgroups: good (5 or 4), average (3), and poor (2 or 1). Chronic diseases such as hypertension, diabetes, heart disease, and stroke were coded as dichotomous variables based on whether the subjects reported having those diseases and whether they were diagnosed by a physician. Depressive symptoms were measured using a shorter version of the Center for Epidemiologic Studies Depression Scale (CES-D)^27^. Each item was answered on a scale of 0 (rarely or none of the time) to 3 (most or almost all the time), and the total score ranged from 0 to 30, with higher scores indicating greater levels of depressive symptoms.

Health behaviors included smoking, alcohol drinking, and physical activity. Smoking and drinking status was dichotomized as yes or no from the question “Do you smoke/drink currently?”. In our study, physical activity was recoded as a dichotomous variable based on whether the respondents participated in the following activities: (1) joining organized group activities, such as folk dance, ballroom dance, and tai chi; (2) walking, jogging, hiking, or doing other outdoor fitness activities; or (3) planting or engaging in garden work. Respondents that participated in any one of these activities were classified as engaging in physical activity.

Physical function was assessed by measurements of activities of daily living (ADL), instrumental activities of daily living (IADL), and mobility tasks. ADL included bathing, dressing, eating, movement in bed/transferring, ambulating indoors, and toilet use. IADL consisted of shopping, managing money, riding a bus or train independently, doing heavy work around the house or yard, doing light housework, and using the telephone. Mobility tasks comprised standing continuously for 15 minutes, squatting, lifting arms over head, holding or grasping with fingers, lifting or carrying 12 kg, running or jogging 20–30 m, climbing stairs, and walking 200–300 m. Each item was scored from 0 (no difficulty) to 3 (unable to do the activity). The total scores ranged from 0 to 18 for ADL, 0 to 18 for IADL, and 0 to 24 for mobility tasks. The higher scores represented worse functioning.

Social support contained social interaction and emotional support. Social interaction was realized as two variables, playing games (chess, cards, or mahjong) and socializing with friends, neighbors, or relatives. Emotional support comprised respondents being cared for when ill and being listened to by friends or relatives. Each item was scored 0 (no) or 1 (yes), and the overall social support score was calculated based on the scores for social interaction and emotional support.

### Statistical analysis

We utilized the group-based trajectory model to identify the developmental trajectories of cognitive function based on the SPMSQ score. The group-based trajectory model combines the methods for finite mixture models and cluster analysis with longitudinal data. The model classifies individuals into clusters with similar trajectories according to their longitudinal data, assuming that individual differences in trajectories can be summarized by a finite set of different polynomial functions for age or time^28^,^29^. The model allows continuous and discrete values. The SPMSQ with a 5-point scale is an ordinal variable, but it is analyzed as a continuous variable at the manifest level. This model was analyzed by using the PROC TRAJ macro in SAS^30^,^31^ (http://www.andrew.cmu.edu/user/bjones/index.htm). PROC TRAJ has an option for censored normal. We applied it and set the minimum to 0 and maximum to 5 for SPMSQ. PROC TRAJ uses the maximum likelihood method for the estimation. It uses all the individuals in the data set, including those with missing values. However, only available data for each individual are incoporated in the analysis. In other words, individuals with partially missing data were not removed from the analysis. The optimal number of groups was determined by the Bayesian Information Criterion^29^.

Baseline characteristics were expressed as mean and standard deviation (SD) for continuous variables and proportions for categorical variables. Chi-squared test (for categorical variables) and analysis of variance (for continuous variables) were used to compare the differences among the trajectory groups. Multinomial logistic regression (MLR) was applied to identify baseline variables, including demographic, physical, mental, and social predictors of group membership. Least squares means (LSM) of SPMSQ adjusting for demographic characteristics were calculated, and Bonferroni correction was applied in the comparisons of SPMSQ score over time among the trajectory groups^32^. We finally used a mixed model to assess the longitudinal variation of physical, mental, and social variables among the trajectory groups. All analyses were implemented using SAS statistical software (version 9.3 for Windows; SAS Institute, Inc., Cary, NC), and the alpha level was set at 0.05.

## Results

### Development patterns of cognitive impairment

**Figure 2:**
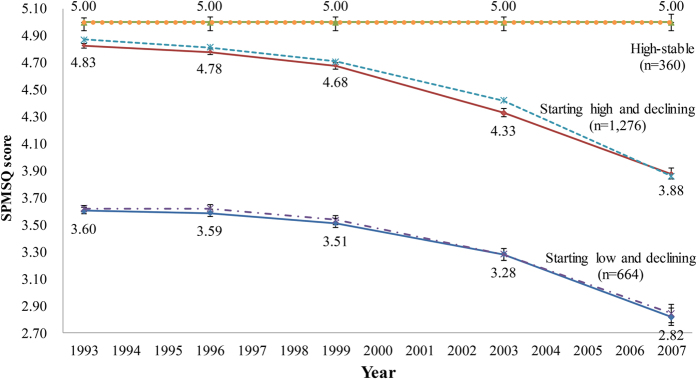
Trajectories of cognitive change among 2300 Taiwanese elderly individuals, 1993–2007, assessed by Short Portable Mental Scale Questionnaire (SPMSQ). The observed trajectory is displayed by a solid line, and the predicted trajectory is shown by a dashed line. The SPMSQ score was modified by Least Squares Means, and there was significant interaction between SPMSQ score and year (p < 0.001). *Significant difference in comparison to the 1993 survey in each group after Bonferroni adjustment (p < 0.0125). ^†^Significant difference in comparison with the high-stable group in each survey after Bonferroni adjustment (p < 0.025).

Figure 2 presents three trajectory groups based on the Bayesian Information Criterion (BIC) value as the best model (−9237.24 for the number of participants; −9245.01 for the number of observations). Figure 2 shows both the actual trajectory (solid line) and the predicted trajectory (dashed line). These three groups did not cross over during the follow-up time. The average posterior probability of the three groups ranged from 0.74 (SD = 0.13) to 0.89 (SD = 0.14), indicating adequate internal reliability on the basis of the 0.7 threshold of posterior probability recommended by Jones and Nagin^31^. The three groups were labeled as (1) starting low and declining, which represented 664 subjects (30.8%) who had a lower score that continued to decrease over time; (2) starting high and declining, which showed a declining trend from a high score to a lower score and included 1276 of the subjects (51.8%); and (3) high-stable, which included 360 of the subjects (17.4%), who maintained a high score of 5 over time.

We used LSM to calculate the adjusted SPMSQ score, and used Bonferroni correction to compare the differences among trajectories. After adjustment for the socio-demographic variables, the SPMSQ score varied across time in comparison to the initial one in the starting high and declining group and the starting low and declining group (Fig. 2). Compared with the high-stable group, the starting high and declining group and starting low and declining group had significantly lower SPMSQ at each time point (p < 0.025).

### Characteristics at baseline

Table 1 summarizes the baseline characteristics of the three trajectory groups. The mean age of all 2300 participants was 70.95 years (range 65–93), and 44.87% of them were female. All variables except BMI, hypertension, and heart disease were statistically significant among the three groups (p < 0.05). Subjects in the starting low and declining group were more likely to be older, female, and less educated; had a lower proportion taking part in physical activity, smoking, and alcohol drinking; and had poor health and physical functioning, more depression, and less emotional support compared with the other two groups.

### Baseline variables related to the trajectories

After adjustment for age, sex, and education, MLR was used to calculate the relationships between baseline variables and trajectory groups (Table 2). In a comparison of the starting high and declining group and the high-stable group, smoking (odds ratio [OR], 1.35; 95% confidence intervals [CI], 1.01 to 1.84), diabetes (OR, 1.63; 95% CI, 1.01 to 2.99), CES-D (OR, 1.04; 95% CI, 1.01 to 1.07), and IADL (OR, 1.21; 95% CI, 1.03 to 1.42) were significantly different at baseline. The odds ratio of 1.35 for smoking indicated that a person who smoked had 1.35 times higher odds of being in the starting high and declining group relative to the high-stable group than a person who did not smoke. We found that the same variables were significantly related to being in the starting low and declining group; in addition, emotional support (OR, 0.77; 95% CI, 0.60 to 0.99) had a protective effect in the starting low and declining group compared with the high-stable group. In other words, increasing emotional support by 1 point decreased the odds of being in the starting low and declining group by 23%.

### Time-varying variables related to the trajectories

**Figure 3:**
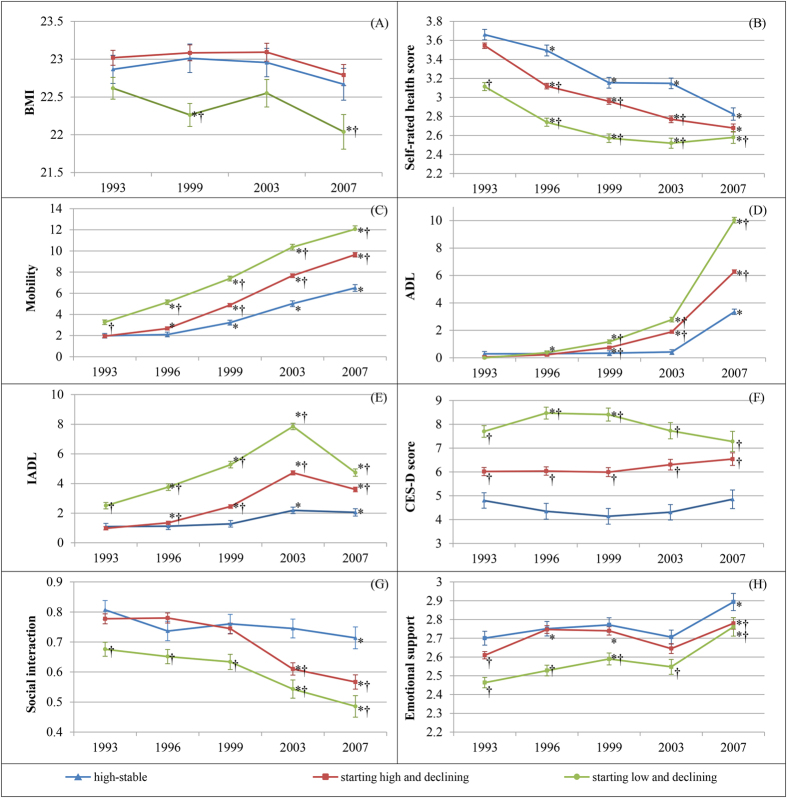
Time trend of continuous variables for each trajectory group. (**A**) BMI; (**B**) Self-rated health score; (**C**) Mobility score; (**D**) ADL score; (**E**) IADL score; (**F**) CES-D score; (**G**) Social Interaction score; (**H**) Emotional support score. *Significant difference in comparison with the 1993 survey in each group after adjusting the socio-demographic variables. ^†^Significant difference in comparison with the high-stable group in each survey after adjusting for the socio-demographic variables.

**Figure 4:**
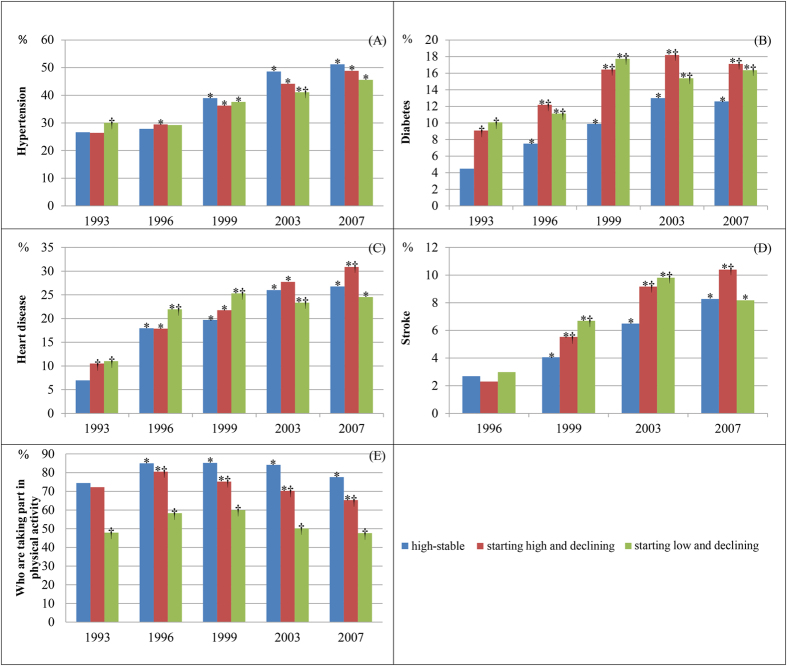
Time trend of categorical variables for each trajectory group. (**A**) Proportion with hypertension; (**B**) proportion with diabetes; (**C**) proportion with heart disease; (**D**) proportion with stroke; (**E**) proportion taking part in physical activity. *Significant difference in comparison to the 1993 survey in each group after adjusting the socio-demographic variables. ^†^Significant difference in comparison with the high-stable group in each survey after adjusting for the socio-demographic variables.

To account for the repeated measures, we used a mixed model to examine the association between the time-varying variables and the trajectories, adjusting for socio-demographic variables (Figs 3 and 4). Figure 3 shows the time trend of BMI, self-rated health score, physical function score, depression score, and social support score for each trajectory group. Figure 4 displays the time trend in the proportion of cardiovascular diseases for the trajectories. The majority of the variables appear to have the same variation among the three trajectory groups. Most of the variables varied across time, especially in the two decline groups. The time and group interaction also existed for almost all variables, with the exceptions of BMI and CES-D score. Subjects who rated their health as poorer and had impaired physical function, worse depression symptoms, less emotional support, less physical activity, and greater likelihood of diabetes, heart disease, or stroke were more likely to be in the starting high and declining group than the high-stable group, after time, time-group, sex, age, and education levels were accounted for similar patterns were observed in the starting low and declining group.

## Discussion

This study utilized a longitudinal follow-up of elderly subjects to identify the developmental trajectories of cognitive impairment and found three trajectories with no crossover during the follow-up time. The trajectories begin prior to 65 years of age. Elderly subjects who smoked, did not drink alcohol, or had depression, diabetes, or poor physical function at baseline tended to be clustered into the starting high and declining and starting low and declining groups. Physical functioning, depression, cardiovascular diseases, social support, and physical activity were related to the progression of the decline in cognitive function.

We found that elderly subjects who were smokers at baseline were more likely to have a decline in cognitive score over time^33^. This finding is compatible with previous observations that smoking increases the risk of cognitive dysfunction. However, one study found that current smokers and ever smokers may be less likely to suffer from cognitive impairment than nonsmokers^16^. This outcome could reflect a survival effect; that is, some smokers might have died prematurely, and those who survived might have had better health.

Depression is a major risk factor for the incidence of cognitive impairment^34^, which our baseline and longitudinal results confirmed. Previous studies observed that patients with cognitive impairment who also experience depression had more serious cognitive deficits than those without depression^35^,^36^. Depression is believed to increase the secretion of glucocorticoids, which may impair cognitive function and damage the blood vessels of the frontal lobe or cause degenerative neural disease^37^. In addition, depression may cause a loss of appetite and lead to a decline in physical function, which may lead to lower BMI and further result in cognitive impairment.

Self-rated health condition is an important factor that influences the quality of life and satisfaction^38^ and may even predict mortality^39^. A person with better self-rated health is more motivated to maintain the current status and to engage in more health behaviors^40^. We discovered that elderly subjects with poorer physical activity had poor cognitive function initially and their cognitive function continued to decline over time. Higher levels of physical activity could decrease the occurrence of cognitive dysfunction^15^,^41^ because it could decrease the occurrence of cognitive decline^42^ and increase the size of the hippocampus to improve cognitive abilities^43^. Physical activity might even increase the cognitive reserve to reduce the risk of cognitive decline and decrease the incidence of dementia^44^.

We found that declining cognitive trajectories were significantly associated with physical function. This finding is similar to the study by Auyeung *et al.*^45^, which indicated that poor physical function coexists with cognitive impairment. Worse physical function is hypothesized to increase the risk of cognitive decline^46^ on the basis that both physical and cognitive functions affect each other. Cognitive function is necessary for the performance of certain daily activities, and the performance of these activities can maintain or improve cognitive ability^47^.

Vascular risk factors are associated with having worse cognitive performance^48^. Diabetes was significantly related to a declining cognitive trajectory, illustrating that diabetes may be an important factor affecting elderly people with a decreasing SPMSQ score over time, especially in the high-decline group. Factors related to diabetes, including insulin resistance, glucose toxicity, and inflammation, can lead to different pathologies underlying cognitive impairment^49^. Insulin resistance may affect the coordination of nerves in brain regions and reduce or block blood flow to the brain^50^. Glucose toxicity results in vascular damage and influences the generation of neurodegenerative disorders in brain^51^. Hyperglycemia may cause decrements in working memory and attention^51^ and may cause cognitive impairments^52^. Inflammation is considered to be a factor initiating insulin resistance and the development of diabetes^53^. The association between inflammation and cognitive impairment among the elderly should be studied. Stroke patients appear to have a higher risk of cognitive dysfunction^54^ possibly because stroke may damage the brain and affect the function of memory, leading to cognitive impairment^55^.

A positive relationship between social capital and cognitive function was found in our study, and this outcome corresponds to findings reported by Green *et al.*^56^ and Hughes *et al.*^57^. Social support has been suggested to prevent cognitive decline by regulating mood and social capacity^58^. Elderly individuals may derive social and emotional support from participating in social activities. Social support not only enhances their life satisfaction and well-being, but also avoids isolation from a social network, potentially decreasing the risk of cognitive impairment^57^.

The present study has several limitations. First, all variables were self-reported, thus recall bias is consequently unavoidable. Nevertheless, parts of the questionnaire have been validated, and the interviewers were well trained. We also made spot checks to ensure that the responses were correctly collected. Secondly, cognitive function was assessed by SPMSQ with five questions, which might restrict the results of our study. Many other instruments are available to evaluate cognitive function in the elderly, such as Mini-Mental State Examination (MMSE). MMSE, the most commonly used assessment for cognitive impairment, is a 30-point scale including tests of orientation, registration, attention and calculation, recall, and language^59^. SPMSQ is a screening instrument for cognitive dysfunction rather than a precise test of declines in specific cognitive functions. We only screened a population prior to mild cognitive impairment in our study; therefore, our results on factors of cognitive impairment may not be generalizable to people with severe cognitive impairment and should be interpreted with caution. In addition, the reliability in our study is 0.63, indicating that the quality of the five-item instrument is acceptable. Thirdly, a ceiling effect may exist in the high-stable group. For the scales, a percentage of 20% at ceiling was considered a significant effect^60^. However, participants in high-stable group accounted for only 15.7% of the subjects, and not all of them had perfect score. Therefore, we could ignore the ceiling effect in our results. Finally, the factors influencing the progression of cognitive disorders are complicated. We might not have exhausted all the potential factors, although our results were in accord with those of other researchers.

In conclusion, the development of cognitive impairment trajectories varied. Impairment in the starting low and declining group appeared before 65 years of age. Physical function impairment, diabetes, heart disease, stroke, depression, and poor social capital in late life may be potential risk factors that affect the decline of cognitive function. Prevention and screening should begin early to delay deterioration. Different risk factors were associated with the trajectories of cognitive function, and preventive strategies should be designed accordingly.

## Additional Information

**How to cite this article**: Chen, T.-Y. and Chang, H.-Y. Developmental Patterns of Cognitive Function and Associated Factors among the Elderly in Taiwan. *Sci. Rep.* **6**, 33486; doi: 10.1038/srep33486 (2016).

## Figures and Tables

**Table 1:** Baseline characteristics of the study population by trajectory group in 1993.

Variables	Total (N = 2,300)	Starting low and declining (n = 664)	Starting high and declining (n = 1,276)	High-stable (n = 360)	p-value^a^
Age, y, mean ± SD	70.95 ± 5.04	72.89 ± 5.83	70.63 ± 4.61	68.50 ± 3.26	<0.001
65–74, n (%)	1812 (78.78)	422 (63.55)	1047 (82.05)	343 (95.28)	<0.001
≥75, n (%)	488 (21.22)	242 (36.45)	229 (17.95)	17 (4.72)	
Female, n (%)	1032 (44.87)	473 (71.23)	458 (35.89)	101 (28.06)	<0.001
Education, n (%)					<0.001
Illiterate	883 (38.41)	493 (74.36)	356 (27.90)	34 (9.44)	
Uneducated but literate	185 (8.05)	48 (7.24)	115 (9.01)	22 (6.11)	
Primary	769 (33.45)	104 (15.69)	504 (39.50)	161 (44.72)	
Secondary or above	462 (20.10)	18 (2.71)	301 (23.59)	143 (39.72)	
Health behavior, n (%)					
Take part in physical activity	1507 (65.52)	318 (47.89)	921 (72.18)	268 (74.44)	<0.001
Smoking	667 (29.00)	112 (16.87)	437 (34.25)	118 (32.78)	<0.001
Alcohol drinking	447 (19.43)	57 (8.58)	285 (22.34)	105 (29.17)	<0.001
BMI^b^, kg/m^2^, mean ± SD	22.83 ± 3.36	22.63 ± 3.52	22.93 ± 3.39	22.88 ± 2.96	0.17
Normal	1363 (60.44)	402 (61.75)	745 (59.79)	216 (60.34)	0.17
Underweight	159 (7.05)	50 (7.68)	84 (6.74)	25 (6.98)	
Overweight	480 (21.29)	117 (17.97)	277 (22.33)	86 (24.02)	
Obese	253 (11.22)	82 (12.60)	140 (11.24)	31 (8.66)	
Self-rated health (0–5), mean ± SD	3.44 ± 1.08	3.06 ± 1.10	3.56 ± 1.05	3.71 ± 0.99	<0.001
Good, n (%)	1054 (46.13)	217 (32.93)	636 (50.20)	201 (55.99)	<0.001
Average, n (%)	783 (34.27)	229 (34.75)	432 (34.10)	122 (33.98)	
Poor, n (%)	448 (19.61)	213 (32.32)	199 (15.71)	36 (10.03)	
Chronic disease, n (%)					
Hypertension	616 (27.49)	192 (30.09)	330 (26.40)	94 (26.63)	0.22
Diabetes	193 (8.64)	64 (10.08)	113 (9.08)	16 (4.49)	0.008
Heart disease	207 (10.08)	63 (11.01)	121 (10.51)	23 (6.95)	0.11
CES-D (0–30), mean ± SD	6.31 ± 5.71	8.08 ± 6.67	5.89 ± 5.19	4.52 ± 4.62	<0.001
Physical function, mean ± SD					
Mobility tasks (0–24)	2.39 ± 3.91	4.31 ± 5.05	1.76 ± 3.16	1.11 ± 2.33	<0.001
ADLs (0–18)	0.09 ± 0.76	0.26 ± 1.29	0.02 ± 0.39	0.01 ± 0.17	<0.001
IADLs (0–18)	1.50 ± 2.85	3.28 ± 4.05	0.89 ± 1.89	0.43 ± 1.07	<0.001
Social support, mean ± SD					
Social interaction (0–2)	0.75 ± 0.60	0.63 ± 0.54	0.79 ± 0.61	0.85 ± 0.65	<0.001
Emotional support (0–3)	2.58 ± 0.75	2.49 ± 0.80	2.60 ± 0.76	2.69 ± 0.68	<0.001

SD: standard deviation; BMI: body mass index; CES-D: Center for Epidemiologic Studies Depression Scale; ADL: activities of daily living; IADL: instrument activities of daily living.

^a^Chi-square test or ANOVA were applied to test for differences among the three trajectory groups.

^b^Underweight: BMI < 18.5 kg/m^2^; Normal: 18.5 ≤ BMI < 24 kg/m^2^; Overweight: 24 ≤ BMI < 27 kg/m^2^; Obese: BMI ≥ 27 kg/m^2^.

**Table 2:** Baseline factors associated with trajectory in multinomial logistic regression.

Variables	Starting high and declining^a^	Starting low and declining^a^
	OR (95% CI)	OR (95% CI)
Physical activity	1.17 (0.86 to 1.60)	0.64 (0.43 to 0.93)
Smoking	1.35 (1.01 to 1.84)	1.44 (0.93 to 2.25)
Alcohol drinking	0.77 (0.56 to 1.06)	0.63 (0.39 to 1.01)
BMI	1.00 (0.96 to 1.05)	0.98 (0.93 to 1.04)
Self-rated health status	1.05 (0.90 to 1.22)	0.94 (0.77 to 1.14)
Hypertension	0.90 (0.64 to 1.26)	1.27 (0.82 to 1.97)
Diabetes	1.63 (1.01 to 2.99)	1.82 (0.92 to 3.59)
Heart disease	1.70 (0.97 to 2.97)	1.15 (0.57 to 2.30)
CES-D score (0–30)	1.04 (1.01 to 1.07)	1.05 (1.01 to 1.08)
Mobility tasks score (0–18)	0.97 (0.89 to 1.05)	0.92 (0.84 to 1.00)
ADL score (0–18)	0.83 (0.53 to 1.31)	0.87 (0.56 to 1.36)
IADL score (0–18)	1.21 (1.03 to 1.42)	1.50 (1.27 to 1.78)
Social interaction (0–2)	0.98 (0.78 to 1.23)	0.87 (0.64 to 1.17)
Emotion support (0–3)	0.87 (0.71 to 1.07)	0.77 (0.60 to 0.99)

OR: odds ratio; BMI: body mass index; CES-D: Center for Epidemiologic Studies Depression Scale; ADL: activities of daily living; IADL: instrument activities of daily living.

^a^High-stable group was considered as reference group.

## References

[b1] World Health Organization. Facts about ageing. (2014) Available at: http://www.who.int/ageing/about/facts/en/. (Accessed: 30th June 2015).

[b2] Prince M., Wimo A., Guerchet M. *et al.* World Alzheimer Report 2015: The Global Impact of Dementia-An analysis of prevalence, incidence, cost and trends. (2015) Available at: https://www.alz.co.uk/research/WorldAlzheimerReport2015.pdf. (Accessed: 1st March 2016).

[b3] Dewey M. E. & Saz P. Dementia, cognitive impairment and mortality in persons aged 65 and over living in the community: a systematic review of the literature. Int J Geriatr Psychiatry. 16, 751–761 (2001). 11536341 10.1002/gps.397

[b4] Thies W., Bleiler L. & Alzheimer’s A. 2013 Alzheimer’s disease facts and figures. Alzheimers Dement. 9, 208–245 (2013). 23507120 10.1016/j.jalz.2013.02.003

[b5] Fitzpatrick A. L., Kuller L. H., Lopez O. L., Kawas C. H. & Jagust W. Survival following dementia onset: Alzheimer’s disease and vascular dementia. J Neurol Sci. 229–230, 43–49 (2005). 10.1016/j.jns.2004.11.022 15760618

[b6] Sanders S. Is the glass half empty or half full? Reflections on strain and gain in caregivers of individuals with Alzheimer’s disease. Soc Work Health Care 40, 57–73 (2005). 15837668 10.1300/J010v40n03_04

[b7] Schoenmakers B., Buntinx F. & Delepeleire J. Factors determining the impact of care-giving on caregivers of elderly patients with dementia. A systematic literature review. Maturitas. 66, 191–200 (2010). 20307942 10.1016/j.maturitas.2010.02.009

[b8] Huang S. S., Lee M. C., Liao Y. C., Wang W. F. & Lai T. J. Caregiver burden associated with behavioral and psychological symptoms of dementia (BPSD) in Taiwanese elderly. Arch Gerontol Geriatr. 55, 55–59 (2012). 21601931 10.1016/j.archger.2011.04.009

[b9] Lee S., Colditz G. A., Berkman L. F. & Kawachi I. Caregiving and risk of coronary heart disease in US women-A prospective study. Am J Prev Med. 24, 113–119 (2003). 12568816 10.1016/s0749-3797(02)00582-2

[b10] Taiwan Alzheimer’s Disease Association. Knowing the dementia. (2015) Available at: http://www.tada2002.org.tw/tada_know_02.html. (Accessed: 1st March 2016).

[b11] Kim S., Kim Y. & Park S. M. Association between alcohol drinking behaviour and cognitive function: results from a nationwide longitudinal study of South Korea. BMJ Open. 6, e010494 (2016). 10.1136/bmjopen-2015-010494 PMC4854012 27118285

[b12] Seliger S. L., Wendell C. R., Waldstein S. R., Ferrucci L. & Zonderman A. B. Renal function and long-term decline in cognitive function: the Baltimore Longitudinal Study of Aging. Am J Nephrol. 41, 305–312 (2015). 26201453 10.1159/000430922 PMC4544717

[b13] Wu M. S., Lan T. H., Chen C. M., Chiu H. C. & Lan T. Y. Socio-demographic and health-related factors associated with cognitive impairment in the elderly in Taiwan. BMC Public Health. 11, 22 (2011). 21223555 10.1186/1471-2458-11-22 PMC3027136

[b14] Masi S. *et al.* 4c.01: Lifetime Obesity, Cardiovascular Disease and Cognitive Function: A Longitudinal Study from the 1946 Birth Cohort. J Hypertens 33, Suppl 1:e56 (2015).

[b15] Sun Y. *et al.* A nationwide survey of mild cognitive impairment and dementia, including very mild dementia, in Taiwan. PLoS One 9, e100303 (2014). 24940604 10.1371/journal.pone.0100303 PMC4062510

[b16] Wang C. C. *et al.* Cigarette smoking and cognitive impairment: a 10-year cohort study in Taiwan. Arch Gerontol Geriatr. 51, 143–148 (2010). 19833398 10.1016/j.archger.2009.09.041

[b17] Gunstad J., Lhotsky A., Wendell C. R., Ferrucci L. & Zonderman A. B. Longitudinal examination of obesity and cognitive function: results from the Baltimore longitudinal study of aging. Neuroepidemiology 34, 222–229 (2010). 20299802 10.1159/000297742 PMC2883839

[b18] Blondell S. J., Hammersley-Mather R. & Veerman J. L. Does physical activity prevent cognitive decline and dementia? A systematic review and meta-analysis of longitudinal studies. BMC Public Health. 14, 510 (2014). 24885250 10.1186/1471-2458-14-510 PMC4064273

[b19] Health Promotion Administration, Ministry of Health and Welfare. Taiwan Longitudinal Study on Aging (TLSA). (2015) Available at: http://www.hpa.gov.tw/English/ClassShow.aspx?No=200803270009. (Accessed: 30th June 2015).

[b20] Chang M. & Hermalin A. 1989 survey of health and living status of the elderly in Taiwan: questionnaire and survey design. Comparative Study of the Elderly in Four Asian Countries, Research Report. (1989).

[b21] Pfeiffer E. A short portable mental status questionnaire for the assessment of organic brain deficit in elderly patients. J Am Geriatr Soc. 23(10), 433–441 (1975). 1159263 10.1111/j.1532-5415.1975.tb00927.x

[b22] Katzman R. *et al.* A Chinese version of the Mini-Mental State Examination; impact of illiteracy in a Shanghai dementia survey. J Clin Epidemiol. 41, 971–978 (1988). 3193141 10.1016/0895-4356(88)90034-0

[b23] Herzog A. R. & Wallace R. B. Measures of cognitive functioning in the AHEAD Study. J Gerontol B Psychol Sci Soc Sci. 52, Spec No: 37–48 (1997). 10.1093/geronb/52b.special_issue.37 9215356

[b24] Ofstedal M. B., Zimmer Z. S. & Lin H. S. A comparison of correlates of cognitive functioning in older persons in Taiwan and the United States. J Gerontol B Psychol Sci Soc Sci. 54, S291–S301 (1999). 10542831 10.1093/geronb/54b.5.s291

[b25] Glei D. A. *et al.* Participating in social activities helps preserve cognitive function: an analysis of a longitudinal, population-based study of the elderly. Int J Epidemiol. 34, 864–871 (2005). 15764689 10.1093/ije/dyi049

[b26] Health Promotion Administration, Ministry of Health and Welfare. BMI calculator. Available at: http://health99.hpa.gov.tw/OnlinkHealth/Onlink_BMI.aspx. (Accessed: 30th June 2015).

[b27] Radloff L. S. The CES-D scale: A self-report depression scale for research in the general population. Applied Psychological Measurement. 1, 385–401 (1977).

[b28] Nagin D. S. Analyzing developmental trajectories: A semiparametric, group-based approach. Psychol Methods 4, 139–157 (1999). 10.1037/1082-989x.6.1.18 11285809

[b29] Nagin D. S. & Odgers C. L. Group-based trajectory modeling in clinical research. Annu Rev Clin Psychol. 6, 109–138 (2010). 20192788 10.1146/annurev.clinpsy.121208.131413

[b30] Jones B. L., Nagin D. S. & Roeder K. A SAS procedure based on mixture models for estimating developmental trajectories. Sociol Method Res. 29, 374–393 (2001).

[b31] Jones B. L. & Nagin D. S. Advances in group-based trajectory modeling and an SAS procedure for estimating them. Sociol Method Res. 35, 542–571 (2007).

[b32] Hochberg Y. & Benjamini Y. More powerful procedures for multiple significance testing. Stat Med. 9, 811–818 (1990). 2218183 10.1002/sim.4780090710

[b33] Kalmijn S., van Boxtel M. P., Verschuren M. W., Jolles J. & Launer L. J. Cigarette smoking and alcohol consumption in relation to cognitive performance in middle age. Am J Epidemiol. 156, 936–944 (2002). 12419766 10.1093/aje/kwf135

[b34] Gao Y. *et al.* Depression as a risk factor for dementia and mild cognitive impairment: a meta-analysis of longitudinal studies. Int J Geriatr Psychiatry. 28, 441–449 (2013). 22815126 10.1002/gps.3845

[b35] Pearson J. L., Teri L., Reifler B. V. & Raskind M. A. Functional status and cognitive impairment in Alzheimer’s patients with and without depression. J Am Geriatr Soc. 37, 1117–1121 (1989). 2592718 10.1111/j.1532-5415.1989.tb06674.x

[b36] Richard E. *et al.* Late-life depression, mild cognitive impairment, and dementia. JAMA Neurol. 70, 374–382 (2013). 23599941 10.1001/jamaneurol.2013.603 PMC3694613

[b37] Butters M. A. *et al.* Pathways linking late-life depression to persistent cognitive impairment and dementia. Dialogues Clin Neurosci 10, 345–357 (2008). 18979948 10.31887/DCNS.2008.10.3/mabutters PMC2872078

[b38] Hilleras P. K., Jorm A. F., Herlitz A. & Winblad B. Life satisfaction among the very old: a survey on a cognitively intact sample aged 90 years or above. Int J Aging Hum Dev. 52, 71–90 (2001). 11310575 10.2190/B8NC-D9MQ-KJE8-UUG9

[b39] Lima-Costa M. F., Cesar C. C., Chor D. & Proietti F. A. Self-rated health compared with objectively measured health status as a tool for mortality risk screening in older adults: 10-year follow-up of the Bambui Cohort Study of Aging. Am J Epidemiol. 175, 228–235 (2012). 22193172 10.1093/aje/kwr290

[b40] Pender N. J., Walker S. N., Sechrist K. R. & Frank-Stromborg M. Predicting health-promoting lifestyles in the workplace. Nursing research 39, 326–332 (1990). 2092305

[b41] Buchman A. S. *et al.* Total daily physical activity and the risk of AD and cognitive decline in older adults. Neurology 78, 1323–1329 (2012). 22517108 10.1212/WNL.0b013e3182535d35 PMC3335448

[b42] Sofi F. *et al.* Physical activity and risk of cognitive decline: a meta-analysis of prospective studies. J Intern Med 269, 107–117 (2011). 20831630 10.1111/j.1365-2796.2010.02281.x

[b43] Erickson K. I. *et al.* Exercise training increases size of hippocampus and improves memory. Proc Natl Acad Sci USA 108, 3017–3022 (2011). 21282661 10.1073/pnas.1015950108 PMC3041121

[b44] Churchill J. D. *et al.* Exercise, experience and the aging brain. Neurobiol Aging 23, 941–955 (2002). 12392797 10.1016/s0197-4580(02)00028-3

[b45] Auyeung T. W. *et al.* Functional decline in cognitive impairment–the relationship between physical and cognitive function. Neuroepidemiology. 31, 167–173 (2008). 18784415 10.1159/000154929 PMC2824577

[b46] Black S. A. & Rush R. D. Cognitive and functional decline in adults aged 75 and older. J Am Geriatr Soc. 50, 1978–1986 (2002). 12473009 10.1046/j.1532-5415.2002.50609.x

[b47] Wang L., Larson E. B., Bowen J. D. & van Belle G. Performance-based physical function and future dementia in older people. Arch Intern Med. 166, 1115–1120 (2006). 16717174 10.1001/archinte.166.10.1115

[b48] Snyder H. M. *et al.* Vascular contributions to cognitive impairment and dementia including Alzheimer’s disease. Alzheimers Dement. 11, 710–717 (2015). 25510382 10.1016/j.jalz.2014.10.008 PMC4731036

[b49] Ninomiya T. Diabetes mellitus and dementia. Curr Diab Rep. 14, 487 (2014). 24623199 10.1007/s11892-014-0487-z

[b50] Haan M. N. Therapy Insight: type 2 diabetes mellitus and the risk of late-onset Alzheimer’s disease. Nat Clin Pract Neurol. 2, 159–166 (2006). 16932542 10.1038/ncpneuro0124

[b51] Sommerfield A. J., Deary I. J. & Frier B. M. Acute hyperglycemia alters mood state and impairs cognitive performance in people with type 2 diabetes. Diabetes Care 27, 2335–2340 (2004). 15451897 10.2337/diacare.27.10.2335

[b52] Biessels G. J. *et al.* Place learning and hippocampal synaptic plasticity in streptozotocin-induced diabetic rats. Diabetes 45, 1259–1266 (1996). 8772732 10.2337/diab.45.9.1259

[b53] Doi Y. *et al.* Elevated C-reactive protein is a predictor of the development of diabetes in a general Japanese population: the Hisayama Study. Diabetes Care 28, 2497–2500 (2005). 16186286 10.2337/diacare.28.10.2497

[b54] Barba R. *et al.* Poststroke dementia: clinical features and risk factors. Stroke. 31, 1494–1501 (2000). 10884443 10.1161/01.str.31.7.1494

[b55] Colcombe S. J. *et al.* Cardiovascular fitness, cortical plasticity, and aging. Proc Natl Acad Sci USA 101, 3316–3321 (2004). 14978288 10.1073/pnas.0400266101 PMC373255

[b56] Green A. F., Rebok G. & Lyketsos C. G. Influence of social network characteristics on cognition and functional status with aging. Int J Geriatr Psychiatry. 23, 972–978 (2008). 18449952 10.1002/gps.2023 PMC2650477

[b57] Hughes T. F., Andel R., Small B. J., Borenstein A. R. & Mortimer J. A. The association between social resources and cognitive change in older adults: evidence from the Charlotte County Healthy Aging Study. J Gerontol B Psychol Sci Soc Sci. 63, P241–P244 (2008). 18689766 10.1093/geronb/63.4.p241

[b58] Devanand D. P. *et al.* Depressed mood and the incidence of Alzheimer’s disease in the elderly living in the community. Arch Gen Psychiatry. 53, 175–182 (1996). 8629893 10.1001/archpsyc.1996.01830020093011

[b59] Folstein M. F., Folstein S. E. & McHugh P. R. “Mini-mental state”. A practical method for grading the cognitive state of patients for the clinician. J Psychiatr Res. 12, 189–198 (1975). 1202204 10.1016/0022-3956(75)90026-6

[b60] van der Putten J. J., Hobart J. C., Freeman J. A. & Thompson A. J. Measuring change in disability after inpatient rehabilitation: comparison of the responsiveness of the Barthel index and the Functional Independence Measure. J Neurol Neurosurg Psychiatry. 66, 480–484 (1999). 10201420 10.1136/jnnp.66.4.480 PMC1736299

